# HIV-1 Vpr: a multifunctional accessory protein with potential contributions to AIDS pathogenesis and persistence

**DOI:** 10.3389/fmicb.2026.1971957

**Published:** 2026-10-19

**Authors:** Juan Du, Qingchen Li, Shao-Hua Wang, Yifei Zhao, Ke Zhao

**Affiliations:** 1 Institute of Virology and AIDS Research, First Hospital of Jilin University, Changchun, China; 2 Center of Infectious Diseases and Pathogen Biology, First Hospital of Jilin University, Changchun, China

**Keywords:** AIDS pathogenesis, host-viral interactions, human immunodefciency virus (HIV), long-term non-progressor (LTNP), viral enhancement, viral protein R (Vpr)

## Abstract

Human immunodeficiency virus type 1 (HIV-1) infection can progress to acquired immune deficiency syndrome (AIDS), which is characterized by a rapid decline in CD4^+^ T lymphocyte counts, severe impairment of host immune competence, and recurrent opportunistic infections. Over the past four decades, HIV infection has remained one of the major threats to global public health. Viral protein R (Vpr) is one of nine proteins encoded by HIV RNA genome. Despite consisting of merely 96 amino acids, Vpr exerts multiple functions throughout viral infection and replication and is potentially associated with the progression of AIDS. In this review, we summarize the structural features and biological functions of Vpr, and focus on its multifaceted roles, including the induction of G2/M cell-cycle arrest and apoptotic cell death, facilitation of HIV-1 infection and replication via Env enhancement, modulation of cellular signaling cascades and host gene expression, and participation in AIDS progression. We also include Vpr-mediated regulation of human endogenous retroelement activity and its potential impacts on cellular innate immune responses. This work aims to provide novel analytical perspectives and research directions for in-depth elucidation of Vpr functions and their contributions to AIDS pathogenesis.

## 1 Introduction

In 1981, a disease later designated acquired immune deficiency syndrome (AIDS) was first documented among men who have sex with men (Bekker et al., 2023). Affected patients presented opportunistic infections as well as rare malignancies such as Kaposi's sarcoma (Friedman-Kien, 1981). Causative pathogens were subsequently isolated and formally named human immunodeficiency viruses (HIVs) (Barré-Sinoussi et al., 1983; Gallo et al., 1983). Follow-up virological characterizations established that HIVs belong to *Retroviridae lentiviruses*. As RNA viruses, their replication cycle relies on reverse transcription to generate viral DNA intermediates (Hu and Hughes, 2012).

The RNA genome of HIV-1 generally exceeds 9,000 nucleotides in length. Phylogenetic profiling of viral genomes distinguishes two major HIV lineages, HIV type 1 and HIV type 2 (HIV-1 and HIV-2), alongside dozens of subtypes and circulating recombinant forms (CRFs) (Robertson et al., 2000). Despite extensive genetic subdivision, all HIV genomes share a conserved genetic architecture encoding three distinct sets of viral proteins: (1) three structural proteins, namely Gag, Pol (expressed as the Gag-Pol polyprotein), and Env, which are indispensable for *de novo* HIV virion assembly; (2) two regulatory proteins, Tat and Rev, that enable robust viral transcription and gene expression, and; (3) four accessory proteins, Vif, Vpr, Nef, and Vpu specific to HIV-1 (while most HIV-2 isolates express Vpx instead of Vpu) (Skar et al., 2011), that counteract host antiviral restriction factors, rendering them essential for viral replication and pathogenicity *in vivo* (Collins et al., 2014).

Viral protein R (Vpr) was also expected to neutralize cellular anti-HIV-1 defenses. The *vpr* gene and its protein product were initially characterized in 1987 (Wong-Staal et al., 1987). Vpr is a 96-amino-acid polypeptide that exhibits high sequence conservation across HIV strains (Figure 1A). Subsequent work verified that Vpr is packaged into HIV-1 virions (Paxton et al., 1993), implying that Vpr functions since the viral capsid core entering host cells. It was soon found that Vpr facilitates HIV-1 replication in macrophages (Vodicka et al., 1998; Eckstein et al., 2001). Meanwhile, other investigations demonstrated that Vpr augments HIV-1 infection in CD4^+^ T cells (Goh et al., 1998; Gummuluru and Emerman, 1999; Zhou et al., 2012). Furthermore, Vpr has long been recognized to play an important role in AIDS progression (Wang et al., 1996; Lum et al., 2003). Together, these lines of evidence highlight the essential role of Vpr in HIV-1 pathogenesis. Nevertheless, accumulated functional studies have yielded inconsistent and confusing observations. Multiple biological activities have been ascribed to Vpr, including induction of G2/M cell-cycle arrest, initiation of apoptotic cell death, and modulation of host signaling cascades and gene transcription. To date, however, none of these functions have been definitively validated as drivers of HIV-1 infection or AIDS pathogenesis.

**Figure 1:**
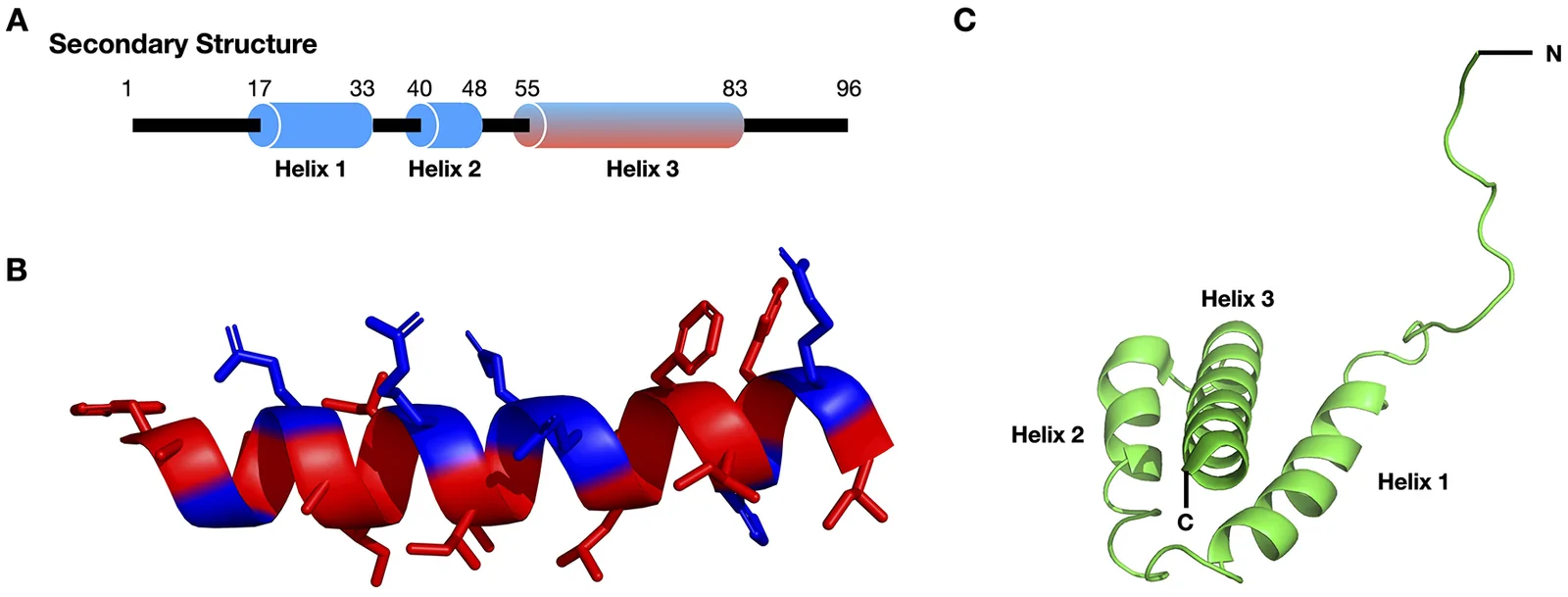
Illustration of Vpr structure and critical residues. **(A)** The secondary structure of the 96-amino-acid-long Vpr protein contains three helices. Helix 1 and 2 are basically hydrophilic (blue), while helix 3 is amphiphilic, with most hydrophilic residues (blue) on one side and hydrophobic one (red) on the other. **(B)** The hydrophilicity of Vpr Helix 3. Hydrophilic (blue) and hydrophobic (red) residues are labeled on the reported structure of Vpr Helix 3. **(C)** The remodeled x-ray-based crystal structure of Vpr (residues 1-74) in the DDB1-DCAF1-Vpr-UNG2 complex. Only Vpr is shown. Vpr structures shown in panels B and C were remodeled based on previously reported structure (PDB: 5JK7) (Wu et al., 2016), with PyMol (version 3.1.0).

## 2 The *vpr* gene and encoded Vpr protein

The *vpr* gene displays striking sequence conservation not only among circulating HIV-1 isolates but also across divergent primate lentiviruses (Fletcher et al., 1996; Planelles et al., 1996; Tristem et al., 1998). Notably, loss-of-function Vpr mutants readily revert to full-length functional Vpr during long-term viral transmission in primates or human (Goh et al., 1998). This observation underscores intense evolutionary selective pressure acting on the *vpr* gene, demonstrating that Vpr function is irreplaceable for sustained HIV-1 transmission *in vivo*.

Structural investigations of Vpr have predominantly utilized nuclear magnetic resonance (NMR) spectroscopy (Wecker et al., 2002; Morellet et al., 2003; Byeon et al., 2021). Three α-helical domains have been mapped to amino acid residues 17–33, 40–48 and 55–83, respectively (Figure 1A). Hydrophilicity profiling of residues within these helices reveals that helices 1 and 2 are largely polar. In contrast, helix 3 exhibits an amphipathic architecture (Figure 1B). Alternative hydrophilic and hydrophobic residues grants this helix the ability to lay on a hydrophobic surface, like the plasmic membrane. This structural feature aligns with earlier immunofluorescence data documenting perinuclear Vpr localization within host cells (Lu et al., 1993).

A high-resolution crystal structure of full-length Vpr has also been resolved together with DDB1- and Cul4- associated factor 1 (DCAF1), DNA damage-binding protein 1 (DDB1), and Uracil-DNA glycosylase 2 (UNG2) (Wu et al., 2016). Consistent with NMR-derived topologies, the resolved Vpr 1-74 folds into three helices within this ternary complex (Figure 1C). Helices 1 and 3 engage DCAF1, which forms a ubiquitin ligase termed cullin-RING ligase 4 (CRL^Cul4^) together with DDB1 and cullin4 (Cul4). Notably, in CRL^Cul4^ complex, Cul4A and Cul4B are interchangable at least for known functions of Vpr (Sharifi et al., 2014). Helix 2 mediates physical contact with UNG2, which is targeted by Vpr for degradation (BouHamdan et al., 1996; Ahn et al., 2010). This structural model delineates the molecular mechanism through which Vpr hijacks host CRL^Cul4^ complexes to execute its biological functions, and further identifies additional role for helix 3 in addition to attaching Vpr to the membrane.

## 3 The functions of Vpr

To achieve optimized viral replication, proteins encoded by HIV-1 frequently exert multiple functions to target diverse intracellular mechanisms. Vpr is no exception; its biological functions include induction of cell-cycle arrest, triggering of apoptosis, modulation of intracellular signaling pathways, and promotion of viral replication in macrophages, etc. A substantial subset of these functions relies on Vpr-mediated hijacking of the cellular ubiquitin-proteasome system (UPS), whereas others are UPS-independent (Figure 2). It is important to note that the current understanding of Vpr functions is primarily built on evidence from various experimental systems. While “*in vitro*” cell line studies provide foundational molecular insights, their physiological relevance to “*in vivo*” HIV-1 infection requires careful evaluation. Significant discrepancies often exist between different experimental models (e.g. tumor cell lines vs. primary cells, overexpression systems vs. viral infection); therefore, the interpretation of the following functions necessitates a clear distinction regarding evidence sources and their biological relevance.

**Figure 2:**
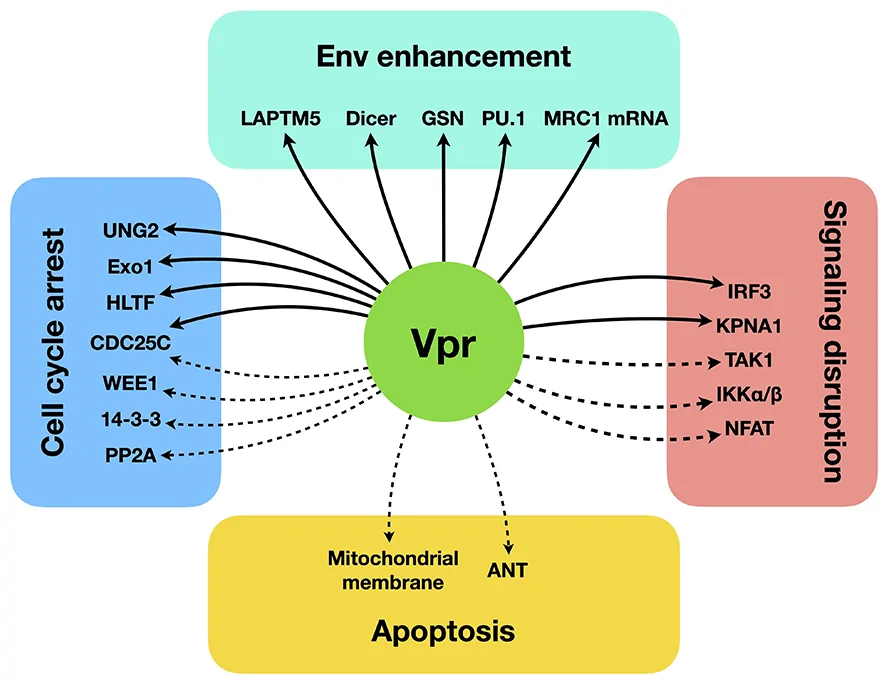
UPS-dependent and independent mechanisms for Vpr functions. Functions of Vpr and associated targets discussed in this review are shown. Solid lines indicate UPS-dependent depletions, while dotted lines indicate UPS-independent pathways. Notably, Vpr does not induce a direct proteasomal depletion of *MRC1* mRNA, but Vpr-mediated *MRC1* mRNA downregulation requires a proteasomal degradation of a yet-unknown factor.

### 3.1 Cell cycle regulation

Shortly following its discovery, Vpr was identified as a potent inducer of G2/M cell-cycle arrest (He et al., 1995; Jowett et al., 1995; Re et al., 1995) (Figure 2). This activity was observed in multiple tumor cell lines and peripheral blood mononuclear cells (PBMCs), and partially accounts for Vpr-mediated growth suppression of these cells. This phenotype has been hypothesized to contribute to HIV-1-associated CD4^+^ T-cell depletion, which however requires further validation with clinical samples. Subsequent investigations established that Vpr triggers G2/M arrest via UPS. Specifically, Vpr hijacks the CUL4-DDB1-DCAF1 ubiquitin ligase complex (i.e., CRL^Cul4^) and acts as a molecular adaptor to recruit target host substrates for proteasomal degradation (Belzile et al., 2007; Tan et al., 2007; Wen et al., 2007). Site-directed single-point mutagenesis studies confirm that physical interaction between Vpr and DCAF1 is indispensable for Vpr-dependent G2/M arrest (Vanegas-Torres and Schindler, 2024). Accordingly, DCAF1 is also referred to as Vpr-binding protein (VprBP).

Screening for UPS substrates depleted by Vpr has uncovered multiple host DNA damage repair factors, including UNG2 (BouHamdan et al., 1996), exonuclease 1 (Exo1) (Yan et al., 2018) and helicase-like transcription factor (HLTF) (Hrecka et al., 2016; Lahouassa et al., 2016). The G2 checkpoint normally halts mitotic entry for cells carrying damaged genomic DNA. By eliminating core DNA repair machinery, Vpr compromises host genome stability, which activates endogenous checkpoint signaling to enforce G2-phase arrest. This signaling cascade includes activation of several kinases like ATR and downstream Chk1/2, leading to inhibitory phosphorylation of CDC25C (short for cell division cycle 25C). Reduced CDC25C phosphatase activity diminishes the kinase function of cyclin B1-cyclin-dependent kinase 1 (CCNB1-CDK1), ultimately locking cells at the G2/M phase.

Complementary UPS-independent pathways also participate in Vpr-driven G2/M arrest. Goh et al. demonstrated direct physical association between Vpr and CDC25C, which impairs the latter's phosphatase catalytic activity (Goh et al., 2004). Furthermore, multiple upstream CDC25C regulators, including WEE1 kinase (Kamata et al., 2008), 14-3-3 scaffold proteins (Kino et al., 2005) and PP2A phosphatase (Elder et al., 2001), interact with Vpr and such interactions are involved in Vpr-induced G2 arrest. Collectively, CDC25C is likely the central effector for Vpr to stall cell cycle.

G2/M arrest confers a replicative advantage to HIV-1, as the 5′ long terminal repeat (5′-LTR), which harbors the proviral promoter, exhibits enhanced transcriptional activity in G2-arrested cells (Goh et al., 1998). Consistent with this model, Vpr boosts HIV-1 replication in proliferating cell lines and primary CD4^+^ T cells through G2/M arrest induction, but not in terminally differentiated macrophages that constantly rest in the G0 phage (Yan et al., 2019; Zhang and Bieniasz, 2020; Gummuluru and Emerman, 1999). Intriguingly, HIV-1 Vif also contributes modest G2/M arrest by targeting PPP2R5, a regulatory subunit governing PP2A phosphatase activity, for proteasomal turnover (Sakai et al., 2006; Salamango et al., 2019). However, Vif exhibits weaker arrest-inducing capacity relative to Vpr (Sakai et al., 2006), and this activity of Vif is not conserved even across isolates within a single HIV-1 subtype (Zhao et al., 2015). These comparative data indicate that Vpr-mediated G2/M arrest alone is sufficient to promote HIV-1 replication, permitting Vif to evolve specialized auxiliary functions such as APOBEC3H degradation (Zhao et al., 2015).

### 3.2 Apoptosis

Beyond suppressing cellular proliferation via G2/M arrest, Vpr initiates apoptotic death in infected cells even under conditions of *de novo* viral replication blockade. This was first observed in cultured Jurkat cells (Hrimech et al., 1999) and later confirmed in PBMCs (Muthumani et al., 2002), suggesting such a process might drive peripheral CD4^+^ T-cell loss (Figure 2). Upon virion delivery into target cells, Vpr permeabilizes the outer mitochondrial membrane, induces the release of pro-apoptotic factors and dissipates mitochondrial membrane potential (Andersen et al., 2008; Li et al., 2009). This cytotoxic effect maps to the C-terminal Vpr fragment spanning residues 52–96, which contains amphipathic helix 3 capable of partial insertion into lipid bilayers such as the mitochondrial outer membrane. Mitochondrial permeabilization releases apoptogenic factors including apoptosis-inducing factor (AIF) and subsequently cytochrome c. Cytosolic cytochrome c facilitates activation of caspase-9, triggering nuclear apoptosis (Budihardjo et al., 1999).

Vpr also elevates intracellular reactive oxygen species (ROS) levels through direct mitochondrial perturbation (Ivanov et al., 2016). Adenine nucleotide translocase (ANT) constitutes a core pore-forming subunit of the mitochondrial permeability transition pore (mPTP) (Halestrap and Brenner, 2003). Vpr binds ANT to disrupt mitochondrial homeostasis, triggering oxidative stress and robust ROS production (Arunagiri et al., 1997; Macreadie et al., 1997; Halestrap and Brenner, 2003). Excess ROS leads to ATR-Chk1-dependent DNA damage signaling and repair deficiency, promoting apoptotic cell death (Ryan et al., 2016; Chisompola et al., 2025).

Vpr's dual capacity to promote viral replication and drive apoptosis creates an evolutionary dilemma for HIV-1 transmission. G2/M arrest upregulates proviral transcription and virion production, favoring spread to neighboring cells *in vivo*. Paradoxically, concurrent apoptosis limits sustained virion output in theory by eliminating infected producer cells before peak viral release. Notably, helix 3 is required for both Vpr functions: DCAF1 binding to induce arrest, and mitochondrial membrane insertion to initiate apoptosis. This structural interdependence implies the two activities are evolutionarily coupled. Multiple laboratories have established a temporal sequence between the two phenotypes: G2 arrest precedes and peaks prior to robust apoptotic onset (Poon et al., 1997; Stewart et al., 1997, 2000). Vpr thus balances proviral amplification and cellular cytotoxicity, though the molecular details governing this equilibrium remain incompletely characterized.

### 3.3 Env expression/stability enhancement

The envelope protein (Env) of HIV-1 is an essential viral protein for the production and release of infective virions, while Vpr utilizes mutiple mechanisms to enhance Env expression or stability. Interestingly, although such a function is observed in various types of HIV-1-targeted cells, most associated studies were conducted in macrophages. For instance, Mashiba et al. once reported that DCAF1 binding is indispensable for Vpr-mediated upregulation of Env in primary macrophages (Mashiba et al., 2014), suggesting that Vpr-induced protein depletion plays an important role in enhancing Env protein levels. Besides, Vpr inhibits the endosome reticulum associated degradation (ERAD) of HIV-1 Env via lysosomal depletion, not only in MDM, but also in monocyte-derived dendritic cells (MDDC) and certain T cell lines (Zhang et al., 2016). Zhao et al. later reported that Vpr drives CRL^Cul4^-dependent proteasomal degradation of lysosomal transmembrane protein 5 (LAPTM5). Loss of LAPTM5 abrogates lysosomal clearance of viral Env glycoprotein, enhancing the infectivity and intercellular transmission of nascent HIV-1 virions among macrophages (Zhao et al., 2021). This at least partially explains the involvement of both DCAF1 and lysosomes in Vpr-mediated upregulation of Env. In addition, virion-delivered Vpr targets endonuclease Dicer for proteasomal depletion as a post-entry event, relieving Dicer-mediated restriction of HIV-1 replication in monocyte-derived macrophages (MDM) (Klockow et al., 2013). Vpr also induces the removal of host factors like gelsolin (GSN) and transcription factor PU.1 (PU.1) via UPS in MDM, which also enhances Env expression (Fabryova et al., 2023; Virgilio et al., 2024) (Figure 2).

Furthermore, Vpr downregulates cell-surface mannose receptor (MR) expression in macrophages to stabilize Env (Lubow et al., 2020). MR was initially characterized as a macrophage surface receptor that captures HIV-1 particles and mediates virion transfer to CD4^+^ T cells (Nguyen and Hildreth, 2003). Paradoxically, MR levels in macrophages decline following HIV-1 infection, indicating MR possesses a post-infection yet negative role against HIV-1 replication at post-entry stages. Follow-up studies revealed that MR destabilizes HIV-1 Env glycoprotein, impairing virion assembly and release from infected macrophages. To neutralize this host restriction, HIV-1 employs both Vpr and Nef to suppress MR protein abundance (Sukegawa et al., 2018; Vigerust et al., 2005). Intriguingly, Vpr induces the proteasomal degradation of a yet unkown factor to reduce levels of *MRC1* mRNA encoding MR. Consequently, Vpr- (and Nef-) dependent MR depletion augments macrophage-to-T-cell HIV-1 transmission efficiency (Lubow et al., 2020) (Figure 2).

In addition to CD4^+^ T lymphocytes, macrophages represent a major target cell population for HIV-1. Elevated Env expression plays multiple roles in HIV-1 infection in macrophages. First, as noted earlier, Env is essential for the production of infectious viral particles. By increasing Env protein levels, Vpr can efficiently boost the generation of infectious virions and enhance the capacity of the virus to infect macrophages, which may help to sustain HIV-1 replication and cell-to-cell spread within macrophage reservoirs. Second, As central sentinel cells of the innate immune system, macrophages phagocytose foreign pathogens and process antigens for cell-surface presentation (Lubow and Collins, 2020). Notably, compared to direct infection, phagocytosis of HIV-1-infected T cells by macrophages is a more efficient way for HIV-1 to infect macrophages (Baxter et al., 2014). Conversely, although the above phenomenon is Env-independent (Baxter et al., 2014), infected macrophages mediate robust Env-dependent trans-infection of CD4^+^ T cells via cell-surface virion presentation (Groot et al., 2008). Third, in addition to phagocytosis, direct cell-cell contact between infected T cells and uninfected macrophages also promotes Env-mediated plasma membrane fusion and the formation of multinucleated giant cells (Bracq et al., 2017). Such giant cells have been observed in HIV-1 infected patients and are highly virus-productive. Together, these observations highlight cell-to-cell transmission across T-cell and macrophage compartments contributes to HIV-1 dissemination both *in vitro* and *in vivo*, where Vpr-mediated enhancement of Env expression/stability makes important contributions.

### 3.4 Signaling regulation

Vpr functions as a pleiotropic modulator of diverse host intracellular signaling cascades (Figure 2). For instance, Vpr exerts direct regulatory control over HIV-1 mRNA biogenesis. Nuclear factor of activated T cells (NFAT) physically binds the HIV-1 LTR and synergizes with nuclear factor κB (NF-κB) to potentiate proviral transcription (Kinoshita et al., 1997). Under physiological conditions, canonical NFAT activation relies on plasma membrane receptor-triggered intracellular Ca^2+^ flux, which drives NFAT nuclear import. Intriguingly, virion-packaged Vpr bypasses upstream stimulation to activate NFAT, not only in Jurkat cells but also in primary T cells and macrophages, thereby facilitating productive HIV-1 infection (Höhne et al., 2016).

Beyond transcriptional regulation, Vpr remodels innate immune signaling and inflammatory responses. Interferon regulatory factor 3 (IRF3) constitutes a core component of the cellular pathogen-sensing mechanism; this transcription factor is indispensable for type I interferon (IFN) expression and downstream induction of IFN-stimulated antiviral genes (ISGs) (Guo et al., 2026). Multiple lines of experimental evidence demonstrate that Vpr promotes proteasome-dependent IRF3 degradation in both Jurkat T lymphocytes and PBMCs (Okumura et al., 2008).

NF-κB signaling represents another context-dependent target of Vpr with conflicting reported outcomes. Canonical NF-κB signaling robustly upregulates HIV-1 proviral gene expression, whereas non-canonical NF-κB activation is hypothesized to reverse HIV-1 latency (Wong and Jiang, 2021). Nevertheless, the outcome of Vpr-dependent NF-κB modulation remains incompletely resolved and controversial across published work. An early 1997 investigation using primary PBMCs first identified Vpr as a negative regulator of NF-κB-driven transcription (Ayyavoo et al., 1997). Subsequent mechanistic investigation confirmed that this suppressive phenotype is mediated through DCAF1 recruitment (Kogan et al., 2013). A comprehensive 2020 study by Khan et al. further delineated the underlying cascade: virion-delivered Vpr forms a complex with DCAF1 to engage karyopherin-α subunit 1 (KPNA1), consequently blocking nuclear translocation of NF-κB (and IRF3) in CD4^+^ T cells (Khan et al., 2020). Conversely, a separate functional screen evaluating multiple naturally occurring Vpr variants in PBMCs failed to detect measurable NF-κB inhibitory activity derived from HIV-1 Vpr (Hotter et al., 2017).

Different from these suppressive observations, Roux et al. (2000) observed Vpr-induced activation of NF-κB (and NF-IL-6) in not only several cultured cell lines, but also in primary T cells and macrophages. Subsequently, accumulating independent data confirm that Vpr stimulates both canonical and non-canonical NF-κB signaling axes. For instance, Vpr induces phosphorylation of transforming growth factor β-activated kinase 1 (TAK1), an event that initiates canonical NF-κB nuclear translocation in primary PBMCs (Liu et al., 2014). Parallel work in Jurkat T cells validated that Vpr drives phosphorylation of IKKα/β, the shared signaling convergence node coordinating both canonical and non-canonical NF-κB signaling pathways and governing downstream transcription factor activation (Liu et al., 2013; Yu et al., 2020). Additional auxiliary NF-κB stimulatory pathways controlled by Vpr have also been characterized. Liang and colleagues uncovered a functional crosstalk between Vpr-induced G2/M cell cycle arrest and NF-κB nuclear shuttling in HEK293T and HeLa cells (Liang et al., 2015). Most recently, macrophage-based research revealed that Vpr-triggered genomic DNA damage activates ATM kinase signaling, which subsequently promotes nuclear translocation of multiple transcription factors including NF-κB (Sandoval et al., 2024).

The inconsistency observed in Vpr-mediated regulation of the NF-κB signaling pathway is somewhat puzzling. Studies reporting either activation or suppression of the NF-κB pathway have employed similar experimental setups. Both phenomena have been observed with overexpressed and virion-delivered Vpr in cultured cell lines as well as primary T cells or MDMs. Notably, however, Kogan et al. did observe that in macrophages derived from five out of six donors, Vpr induced statistically significant inhibition of NF-κB expression. In macrophages from the remaining donor, by contrast, Vpr caused a two-fold increase in NF-κB promoter activity (Kogan et al., 2013). This suggests that macrophages from different individuals intrinsically differ in their capacity to regulate the NF-κB signaling pathway; such variation may be transient or sustained, and different research groups happened to encounter this variability in their respective studies. Interestingly, according to the literature we have compiled, the discrepancies in Vpr-mediated NF-κB pathway regulation might lie in the mechanisms deployed and proteins targeted. UPS and protein degradation seem important for Vpr suppressing the NF-κB pathway, whereas non-UPS pathways appear to be involved in Vpr's activating effect. More importantly, the target proteins for these two opposing effects are distinct. This appears consistent with the above hypothesis: proteins governing NF-κB signaling may vary in abundance among macrophages from different donors, and Vpr acts consequently to yield divergent regulatory outcomes on NF-κB signaling. It is worth noting that this variability may not be restricted to macrophages; groups have also reported conflicting effects of Vpr on NF-κB signaling in primary T cells (Roux et al., 2000; Khan et al., 2020). The reasons underlying these discrepancies remain to be further explored.

Collectively, although the net outcome of Vpr-mediated NF-κB modulation requires further validation, the integrated signaling disruption by Vpr generally converges toward enhanced HIV-1 proviral transcription and replication.

## 4 AIDS pathogenesis

The above functions of Vpr, which either promote virion production or enhance T cell removal, suggest that this viral protein may contribute to HIV-1 pathogenesis. A subset of HIV-1-positive individuals exhibit delayed or absent AIDS progression, classified as long-term non-progressors (LTNPs). As suspected, multiple non-synonymous Vpr variants are genetically linked to the LTNP phenotype, including Q3R, N16H, T19A, Q65R, F72L/A, R77Q/A, R80A, and R90N/K (Vanegas-Torres and Schindler, 2024). The association between these Vpr mutations and attenuated disease progression strongly implicates Vpr as a critical driver of AIDS pathogenesis, yet the underlying molecular pathways remain unresolved.

The functional consequences of Q65R and R80A mutations are readily interpretable: both substitutions disrupt the Vpr-DCAF1 binding interface, abolishing Vpr's capacity to recruit and hijack CRL^Cul4^ complexes required for nearly all characterized Vpr activities (Dobransky et al., 2024). Nonetheless, Q65 and R80 are not the only two residues responsible for Vpr-DCAF1 interaction. Structural and mutagenesis studies have identified additional critical interface residues including R62, L64, H71 and C76 (Wu et al., 2016; Belzile et al., 2007). Notably, variants bearing substitutions at these residues have not been discovered in LTNP isolates. While such mutations may remain undiscovered in LTNP cohorts, this discrepancy alternatively suggests that CRL^Cul4^ hijacking is not sufficient for Vpr's pathogenic function *in vivo*. Supporting this alternative model, LTNP-associated variants T19A and R90N retain wild-type activity across all known Vpr functions (Hadi et al., 2014).

Residue R77 represents another functionally relevant polymorphic site. The R77Q variant was originally associated with LTNP and reported to exhibit defective apoptotic induction capacity (Lum et al., 2003). A contemporary re-evaluation by Solis-Leal et al., however, demonstrated that Vpr R77Q retains full competence to trigger both G2/M arrest and apoptosis in HUT78 T-lymphoid cells, a typical model for HIV studies (Solis-Leal et al., 2024). This aligns with earlier structural data confirming unimpaired Vpr-DCAF1 interaction for R77Q, consistent with intact CRL^Cul4^ recruitment (Jacquot et al., 2009). Meanwhile, Vpr R77Q is less effective in enhancing tumor necrosis factor (TNF) or interleukin-6 (IL-6) expression in an infection-based assay, possibly due to the relative instability of this Vpr mutant when expressing from a proviral vector (Solis-Leal et al., 2024). Strikingly, even at low expression levels, the R77Q mutant elicits exacerbated cell-cycle arrest and cytotoxicity relative to wild-type Vpr (Solis-Leal et al., 2024). This phenotype provides a novel mechanistic framework linking Vpr sequence variation to LTNP status: viruses encoding R77Q Vpr drive early death of infected producer cells before sufficient progeny virions assemble, limiting sustained viral propagation and disease progression.

Taken together, the above lines of evidence demonstrate a clear association between Vpr and AIDS progression. Nevertheless, the mechanism by which Vpr drives AIDS development remains unclear. The decline in CD4^+^ T cells constitutes a hallmark of AIDS pathogenesis (Bekker et al., 2023). Vpr can indeed slow cellular proliferation by inducing G2/M cell cycle arrest and trigger direct T cell death via apoptosis. It may even boost the spread of progeny viruses by elevating HIV LTR promoter activity and Env expression, which further reduces T cell counts. These observations (other functions list above) have been documented in *in vitro* cell lines as well as primary cells as shown previously. However, on the one hand, to our best knowledge, definitive *in vivo* evidence proving that these functions of Vpr are enough to induce AIDS is still lacking. This is mostly due to the difficulties in manipulating Vpr levels in HIV-1-infected patients as well as the lack of clinically applicable inhibitors specific to certain Vpr function(s). On the other hand, multiple LTNP-associated mutations fail to abrogate the aforementioned functions of Vpr, and no shared functional defects have been identified among these Vpr mutants, suggesting that known activities are not sufficient for Vpr-mediated HIV-1 pathogenicity (Vanegas-Torres and Schindler, 2024). Meanwhile, many functions of Vpr rely on the UPS to varying degrees, yet several LTNP-related Vpr mutants retain the capacity to hijack CRL^Cul4^. This indicates that Vpr's impact on AIDS pathogenesis may arise from the synergistic action of multiple functions.

The unresolved mechanistic link between Vpr variants and the LTNP phenotype also implies additional Vpr activities contribute to AIDS pathogenesis. Notably, recent research has identified several novel functions of Vpr. For example, a 2018 study demonstrated that Vpr induces the UPS-dependent depletion of the methylcytosine dioxygenase TET2 in both cell lines and in primary MDM (Lv et al., 2018). This event mediates epigenetic regulation of interleukin 6 (IL-6) expression (Zhang et al., 2015), a cytokine closely associated with the progression risk of HIV-1 infection (Connolly et al., 2005). Consistently, a very recent clinical cohort study established a correlation between high Vpr detection rate, increased IL-6 levels, and aggravated AIDS pathogenesis in HIV-1-infected individuals (Matsunaga et al., 2024). As mentioned above, the transcription factor PU.1 is also targeted and depleted by Vpr via a UPS-dependent manner (Virgilio et al., 2024). PU.1 ablation not only augments HIV-1 Env expression but also suppresses innate immune responses in MDMs, thereby facilitating HIV-1 dissemination. Furthermore, in CEM-CD4 and primary CD4^+^ T cells, Dupont et al. reported that virion-delivered Vpr introduces a UPS-dependent depletion of SMC5-SMC6 complex localization factor 2 (SLF2) (Dupont et al., 2021). This event protects unintegrated viral DNA and enhances viral production. Although whether Vpr exerts the same regulatory effects in HIV-1-infected patients (rather than *ex vivo*-cultured primary cells) remains to be elucidated, these new functions may partially account for Vpr promoting HIV-1 pathogenesis.

## 5 Suppression of LINE-1 retrotransposition

The unresolved mechanistic link between Vpr variants and the LTNP phenotype implies uncharacterized Vpr activities contribute to AIDS pathogenesis. Recent research has uncovered a regulatory axis through which Vpr modulates replication of long interspersed element-1 (LINE-1 or L1) (Kawano et al., 2018; Iijima et al., 2013). L1 elements belong to the broader retroelement (or retrotransposon) superfamily, DNA fragments capable of self-amplification. The replication of retroelements is termed retrotransposition, a multi-step process encompassing target-site nicking and reverse transcription (Huang et al., 2012; Zhao et al., 2022). Within the human genome, L1 is the only known type of retroelement that can replicate autonomously, enabled by reverse transcriptase activity encoded by the L1 ORF2p protein (Jin et al., 2025; Ghanim et al., 2025).

Notably, retroelements like L1 are believed to share a common ancestor with retroviruses like HIV-1 (Goodier, 2016). Furthermore, cytoplasmic RNA and cDNA intermediates generated during L1 retrotransposition act as endogenous agonists of host innate immune sensing pathways (Zhao et al., 2021). These evolutionary and immunological connections provide a biological rationale for HIV-1-mediated L1 regulation. In 2013, Jones et al. first reported that HIV-1 infection augments L1 retrotransposition in Jurkat T cells in a Vif- and Vpr-dependent manner, while L1 DNA accumulates in HIV-1-infected primary CD4^+^ T cells (Jones et al., 2013). Subsequent work validated that extracellular Vpr enhances L1 replication in murine models and patient-derived primary samples (Iijima et al. 2013). Contrastingly, intracellularly expressed Vpr restricts L1 retrotransposition via direct binding to L1 ORF2p, inhibiting ORF2p-associated reverse transcriptase function (Kawano et al., 2018). Vpr is not the sole HIV-1 accessory protein targeting L1 activity; Nef represses L1 mobility by suppressing L1 promoter transcription and disrupting interactions between L1 RNA and its functional protein cofactors (Wang et al., 2022), indicating L1 retrotransposition as a possible blockage for HIV-1 replication.

Collectively, published data reveal a complexed relationship between HIV-1 and endogenous L1 retroelements (Zhao et al., 2021). Notably, although HIV-1-mediated L1 regulation has been detected in diverse experimental systems such as cultured cell lines, primary CD4^+^ T cells, and clinical samples from infected patients, evidence for the involvement of L1 in AIDS progression is still lacking. In theory, intracellular Vpr and Nef function cooperatively to restrain L1 retrotransposition, which may dampen L1-triggered innate immune activation and create a permissive cellular environment for HIV-1 replication and virion release. Conversely, global HIV-1 infection upregulates L1 activity, suggesting downstream consequences of L1 retrotransposition, such as cell-cycle arrest caused by L1-medaited DNA nicking, may indirectly benefit HIV-1 within infected cells and facilitate intercellular transmission. Dissecting the divergent effects of intracellular vs. extracellular Vpr on L1 mobility might deliver new insights into Vpr's multifaceted role in HIV-1 pathogenic cascades.

## 6 Conclusion

Collectively, existing evidence solidifies Vpr as a multifunctional viral protein that coordinates cell cycle manipulation, mitochondrial cytotoxicity, signaling disruption, reservoir maintenance in macrophages, intercellular viral spread, and modulation of L1 retrotransposition. Many outstanding questions persist: how Vpr calibrates the equilibrium between G2 arrest and apoptosis in distinct primary cell subsets; the net outcome of Vpr-mediated NF-κB regulation and its impact on other Vpr functions; the effect of intracellular vs. intercellular Vpr on L1 activity and subsequent impact on HIV-1 replication; the undetermined or uncharacterized pathway(s) or mechanism(s) linking Vpr mutations to the LTNP phenotype; and whether Vpr's diverse activities can be targeted to slow AIDS progression. Further structural, cellular, and translational research focusing on Vpr's host interaction networks will illuminate unmet therapeutic vulnerabilities, unveiling the details of Vpr as the indispensable missing link connecting HIV-1 infection to the onset and progression of AIDS.
